# Inhibition of Ovarian Cancer Cell Spheroid Formation by Synthetic Peptides Derived from Nectin-4

**DOI:** 10.3390/ijms21134637

**Published:** 2020-06-30

**Authors:** Kristin L.M. Boylan, Rory D. Manion, Heena Shah, Keith M. Skubitz, Amy P. N. Skubitz

**Affiliations:** 1 Department of Laboratory Medicine and Pathology, University of Minnesota, Minneapolis, MN 55455, USA; boyla002@umn.edu (K.L.M.B.); manio031@umn.edu (R.D.M.); shahx194@umn.edu (H.S.); 2 Ovarian Cancer Early Detection Program, University of Minnesota, Minneapolis, MN 55455, USA; 3 Department of Medicine, University of Minnesota, Minneapolis, MN 55455, USA; skubi001@umn.edu; 4 Masonic Cancer Center, University of Minnesota, Minneapolis, MN 55455, USA; 5 Department of Obstetrics, Gynecology, and Women’s Health, University of Minnesota, Minneapolis, MN 55455, USA

**Keywords:** ovarian cancer, spheroids, cell aggregation, Nectin-4, peptides, ascites, cell adhesion

## Abstract

The formation of 3D multicellular spheroids in the ascites fluid of ovarian cancer patients is an understudied component of the disease progression. Spheroids are less sensitive to chemotherapy, in part due to the protection afforded by their structure, but also due to their slower proliferation rate. Previous studies suggest that the cell adhesion molecule Nectin-4 plays a key role in the formation of ovarian cancer spheroids. In this study, we further examined the role of Nectin-4 at early time points in spheroid formation using real-time digital photography. Human NIH:OVCAR5 ovarian cancer cells formed aggregates within 8 h, which further contracted into compact spheroids over 24 h. In contrast, Nectin-4 knockdown cells did not form tightly compacted spheroids. Synthetic peptides derived from Nectin-4 were tested for their ability to alter spheroid formation in two ovarian cancer cell lines. Nectin-4 peptide 10 (N4-P10) had an immediate effect on disrupting ovarian cancer spheroid formation, which continued for over 24 h, while a scrambled version of the peptide had no effect. N4-P10 inhibited spheroid formation in a concentration-dependent manner and was not cytotoxic; suggesting that N4-P10 treatment could maintain the cancer cells as single cells which may be more sensitive to chemotherapy.

## 1. Introduction

With an overall 5 year survival rate of only 50%, ovarian cancer is regarded as the deadliest gynecological malignancy [1]. Despite new research and treatment regimens, long-term survival from ovarian cancer has stagnated [2]. The poor prognosis is attributed to the lack of established screening methods for early stage disease, the lack of overt symptoms when the cancer is limited to the ovaries, and drug resistance in late-stage disease leading to recurrence [3].

During the progression of the disease, ovarian cancer cells are shed from the tumor mass and ascites fluid accumulates within the peritoneal cavity. Ascites fluid serves as a rich source of nutrients and is a suitable microenvironment for ovarian cancer cell proliferation and metastasis [4]. Both single cells and spheroids are shed from tumors and metastasize to sites within the peritoneal cavity through the ascites fluid. Most ovarian cancer research has been performed with single cells, leaving the spheroids an understudied facet of ovarian cancer metastasis and tumor recurrence. It is important to consider the physiology of spheroids, as they are more resistant to a wide range of chemotherapies [5,6]. Their resistance is most likely derived from their structure; the outer cells preventing the access of chemotherapy and other metabolites to the internal cells, which includes a population of quiescent or non-dividing cells [6]. When floating in ascites fluid, spheroids exhibit a decreased cell proliferation. They are able to regain their proliferative abilities upon attaching to tissue [7]. Proliferating cells on the surface of spheroids have access to nutrients and are responsible for cell-to-cell signaling within the densely packed aggregates [8]. Cell–cell adhesion enables cell signaling to take place and plays an important role in spheroid formation.

One of the cell adhesion molecules important in spheroid formation is Nectin-4. Nectin-4 plays a key role in initiating and maintaining the adherens junctions of polarized epithelial cells [9,10]. Its characteristic features include three immunoglobin-like extracellular loops that can be cleaved by proteases and found in serum. It has a single transmembrane domain and a short cytoplasmic region that binds afadin, an anchor protein that links Nectin-4 to actin filaments [10]. While Nectin-4 is largely expressed in the placenta, it is also expressed in smaller amounts in healthy skin, stomach, prostate, and lung tissue [11]. By microarray analysis, Nectin-4 was found to be overexpressed in ovarian cancer tissues [12]. This was validated by RT-PCR and immunohistochemistry using tissues and cell lines [13]. Additional studies showed elevated levels of the cleaved Nectin-4 extracellular domain in serum and ascites fluid from ovarian cancer patients [13,14].

We have previously shown that many human ovarian cancer cell lines express Nectin-4; two of these cell lines, NIH:OVCAR5 and CAOV3, express moderate levels of Nectin-4 [13]. We have also shown that when NIH:OVCAR5 cells are seeded in flat-bottom low-attachment plates, they produce large, tightly compacted spheroids, while CAOV3 cells produce smaller, more dispersed aggregates [15]. In previous studies, we genetically modified these cell lines so that they expressed reduced levels of Nectin-4 and we monitored their ability to form spheroids at a limited number of time points over several days [15].

We hypothesize that Nectin-4 plays a role in the adhesion events that occur early in spheroid formation, when interactions between Nectin molecules on adjacent cells are thought to initiate cell–cell adhesion, which is then followed by the recruitment of cadherins and adherens junction formation [10]. Consequently, in this study, we used digital time-lapse photography to accurately and reproducibly monitor cells at early time points in the formation of 3D spheroids using two human ovarian cancer cell lines. This is the first study to screen synthetic peptides derived from the extracellular domain of Nectin-4 for their ability to alter the formation of spheroids at early time points. These peptide studies allowed the discernment of the regions of Nectin-4 that are important for cell–cell adhesion and ovarian cancer spheroid formation. In addition, the ability of the cells to form spheroids in the presence of the most active peptide was monitored to determine the concentration dependence. A scrambled version of the most active peptide was also tested for its effect on spheroid formation. Finally, the active peptide was tested to rule out cytotoxic effects.

## 2. Results

### 2.1. Nectin-4 Expression Promotes the Formation of Multicellular Aggregate Spheroids

**Figure 1:**
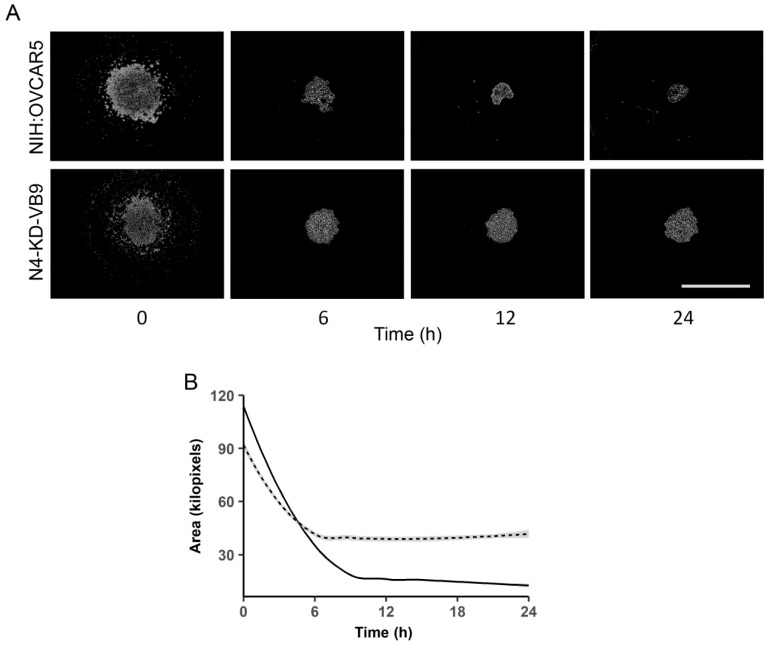
Nectin 4-knockdown cells produce multicellular aggregates that are less compact than parental cells. Round-bottom ultra-low attachment plates were seeded with 5000 cells/well of either NIH:OVCAR5 or NIH:OVCAR5 Nectin-4 knockdown cells (N4-KD-VB9) and allowed to incubate in the gantry of an IncuCyte^®^ Zoom instrument for 24 h. Area of the resulting spheroids was analyzed via CellProfiler^TM^. (**A**) Photographs of the NIH:OVCAR5 cells and the Nectin-4 shRNA knockdown cells (N4-KD-VB9) over a 24 h time period. The initial photographs (t = 0) were taken 10–25 min after the cells were plated, at which time the cells gravitated to the bottom of the well and were initiating cell–cell attachments. Spheroid formation was complete after 24 h. Bar = 800 µm. (**B**) The NIH:OVCAR5 parental cell line, which expresses Nectin-4, contracted into a single tight spheroid over time (solid line), while the N4-KD-VB9 cells that did not express Nectin-4 formed spheroids that were not as tightly compacted (dashed line). These experiments were done in quadruplicate, and the average size was plotted at 15 min intervals over 24 h. Shaded line represents SEM (Ave SEM +/− 0.69 kilopixels for NIH:OVCAR5; +/− 0.72 kilopixels for N4-KD-VB9). Time-lapse videos corresponding to these figures are included as Supplemental videos S1 and S2.

Previous investigations into the role of Nectin-4 revealed a disparity in spheroid formation potential between parental NIH:OVCAR5 cells and Nectin-4 shRNA knockdown cells (N4-KD-VB9) when the cells were monitored at a few selected time points over 3–5 days [15]. Briefly, NIH:OVCAR5 cells plated in a low-attachment environment, in flat bottom wells, produced large, tightly compacted spheroids within 15 h of incubation under standard conditions. Conversely, N4-KD-VB9 cells in a similar environment produced small, loose aggregates; suggesting that Nectin-4 plays an important role in the promotion of spheroid formation. In this study, we expanded upon those earlier findings using high-throughput time-lapse microscopy and computer-aided analysis with an IncuCyte^®^ Zoom instrument to examine the early time points in spheroid formation (Figure 1; Supplemental videos S1 and S2). Figure 1A shows images of spheroid formation over the first 24 h. The first time point (t = 0) was collected 10–25 min after plating 5000 cells per well in round-bottom, ultra-low attachment plates (see Materials and Methods), at which time the cells gravitated to the bottom of the wells and appeared to have initiated cell–cell interactions via Nectin-4 (note that the NIH:OVCAR5 cells, which express Nectin-4, form a larger aggregate than the N4-KD-VB9 cells). After 6 h, both cell lines formed spheroids of similar size. Over the next 18 h, the Nectin-4 expressing NIH:OVCAR5 cells contracted into a compact spheroid, unlike the spheroids formed by the N4-KD-VB9 cells which did not visibly change in size. A graph of spheroid size over time is shown in Figure 1B. The area graphed in Figure 1B represents the sum of the area of the small aggregates (in kilopixels), showing that the NIH:OVCAR5 cells formed compact spheroids within 8 h, while the spheroids formed by the N4-KD-VB9 cells never became compact, even after 24 h of incubation. These data suggest that Nectin-4 is involved in both early adhesion events during spheroid formation, as well as the contraction resulting in the formation of compact spheroids.

### 2.2. Nectin-4 Peptides Inhibit the Formation of Tight Multicellular Aggregates

**Figure 2:**
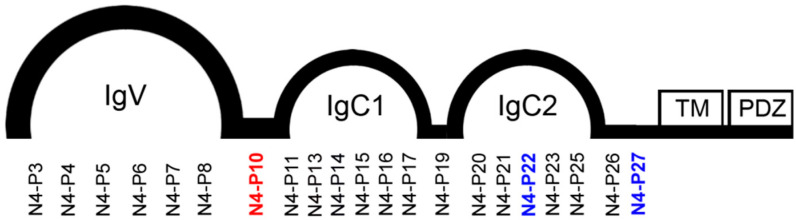
Diagram of the Nectin-4 domains with the associated peptide locations. (Ig = immunoglobulin-like; TM = transmembrane; and PDZ = PDZ binding domain). The location of the three peptides that showed functional activity by inhibiting the formation of spheroids are in bold. The most active peptide is colored red (N4-P10), while the two peptides that were active to a lesser extent are colored blue (N4-P22 and N4-P27).

Twenty-one peptides of 14 amino acids in length encompassing the extracellular domain of Nectin-4 (Figure 2 and Table 1) were synthesized as previously described [15]. The peptides were initially screened at a concentration of 150 µg/mL in 96-well flat-bottom ultra-low attachment plates to identify the section(s) of Nectin-4 that are active in ovarian cancer cell aggregation and spheroid formation. NIH:OVCAR5 cells and peptides were seeded into the plates and manually photographed at 24 and 48 h time points. Three peptides (N4-P10, N4-P22, and N4-P27) significantly impeded the formation of spheroids at both time points (data not shown). However, the cells treated with the other 18 peptides produced spheroids that appeared to be similar in size to the untreated control cells.

**Figure 3:**
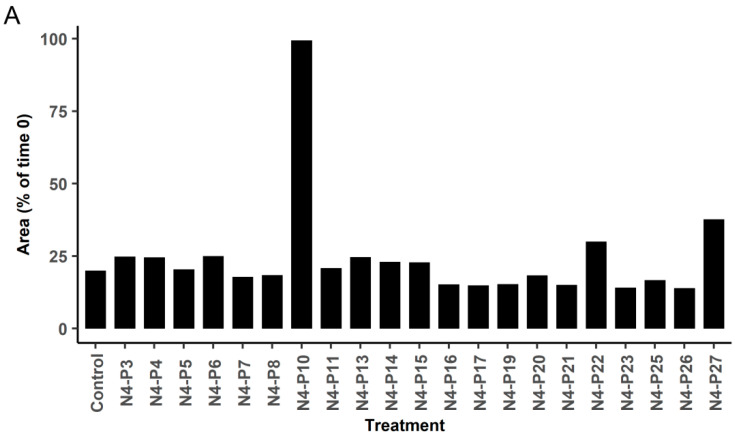

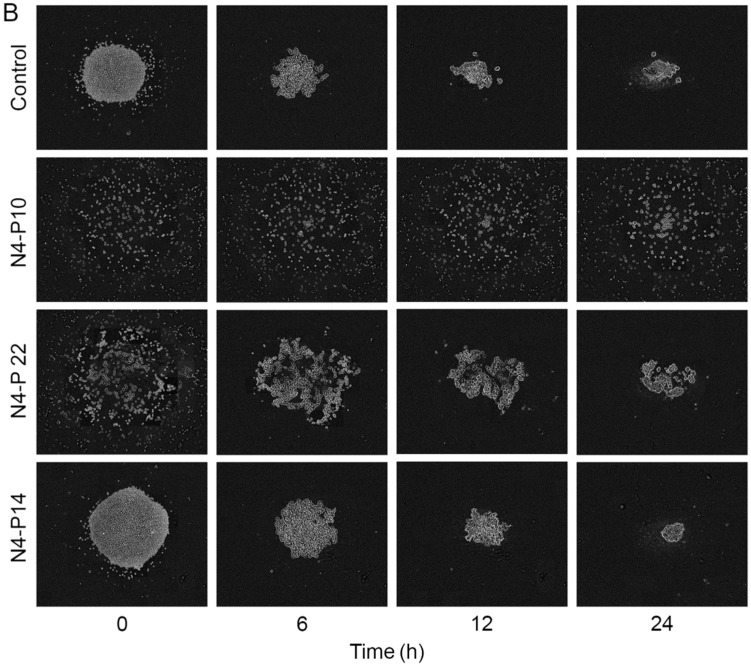
Screening of twenty-one peptides from the extracellular domain of Nectin-4 for their ability to inhibit spheroid formation. Each of the Nectin-4 peptides (at 150 µg/mL final concentration) were added to 96-well round-bottom ultra-low attachment plates followed by the NIH:OVCAR5 cells. The cells were monitored for spheroid formation in the IncuCyte^®^ Zoom instrument and the size of the spheroids was measured. DMSO was used as the negative control. (**A**) Spheroid area is represented as a percentage of the area at 24 h over the area at the time of the initial measurements (*t* = 0). (**B**) Photographs of the NIH:OVCAR5 cells over a 24 h time period in the presence of DMSO (control), or peptides N4-P10, N4-P22, and N4-P14. Bar = 800 µm. The initial photographs (*t* = 0) were taken 10–25 min after the cells were plated, at which time the cells had gravitated to the bottom of the well and were initiating cell–cell attachments. Spheroid formation was complete after 24 h. Time-lapse videos corresponding to these figures are included as Supplemental videos S3–S6.

In order to more accurately quantify the size, shape, and time required for the spheroids to form, the peptides were added to 96-well round-bottom ultra-low attachment plates followed by the addition of NIH:OVCAR5 cells. The cells were monitored in the IncuCyte^®^ Zoom instrument over a 48 h period. The change in spheroid size over the first 24 h of spheroid formation is shown in Figure 3A. Again, peptides N4-P10, N4-P22, and N4-P27 were found to have a significant impact on the formation of spheroids (Figure 3; Supplemental videos S3 and S4), with peptide N4-P10 having the most dramatic effect on spheroid formation. Peptide N4-P22 caused a more modest inhibition of spheroid formation, which was manifested early in the spheroid formation process (Figure 3B; 0 and 6 h time points). This suggests that peptide N4-P22 is less effective at blocking cell adhesion than N4-P10; however, once the cells adhered to each other, the aggregates contracted to form compact spheroids. In stark contrast, the cells treated with the other 18 peptides produced spheroids that were visually and quantitatively indistinct from the DMSO-treated control cells (Supplemental video S5). Peptide N4-P14 is shown as a representative example of a peptide that had no effect on spheroid formation (Figure 3B; Supplemental video S6).

### 2.3. A Scrambled Version of Peptide N4-P10 Did Not Affect Spheroid Formation

**Figure 4:**
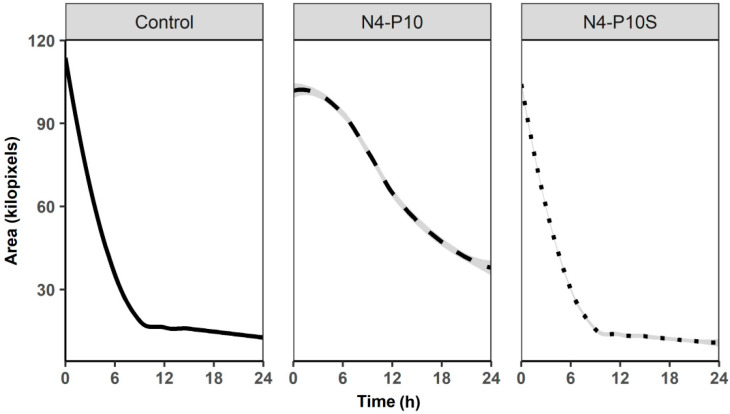
The ability of the Nectin-4 peptide N4-P10 to inhibit spheroid formation depends upon the sequence of the amino acids. DMSO (control) or peptides N4-P10 and N4-P10S (a scrambled amino acid sequence of peptide N4-P10) were added to the wells at a final concentration of 150 µg/mL followed by the addition of the NIH:OVCAR5 cells. The cells were allowed to form spheroids for 24 h. Average spheroid area over time was quantified using a CellProfiler^TM^ workflow. Experiments were done in quadruplicate; shown is the average spheroid area and SEM (shaded line) at each 15 min time point over 24 h (Ave SEM +/− 0.69 kilopixels for Control; +/− 1.92 kilopixels for N4-P10; and +/− 0.92 kilopixels for N4-P10S). A time-lapse video corresponding to the spheroid formation recorded in the presence of peptide N4-P10S is included as Supplemental video S7.

To determine if the activity of peptide N4-P10 was sequence specific, a peptide was synthesized in which the amino acid sequence was scrambled (see Materials and Methods) and tested for the ability to inhibit spheroid formation by the methodology described above. The cells treated with the scrambled peptide N4-P10 (N4-P10S) exhibited a spheroid contraction that was visually and quantitatively similar to that of the untreated controls, while the cells treated with peptide N4-P10 did not form compact spheroids (Figure 4; Supplemental video S7).

### 2.4. Peptide N4-P10 Concentration Dependence and Lack of Cytotoxicity

**Figure 5:**
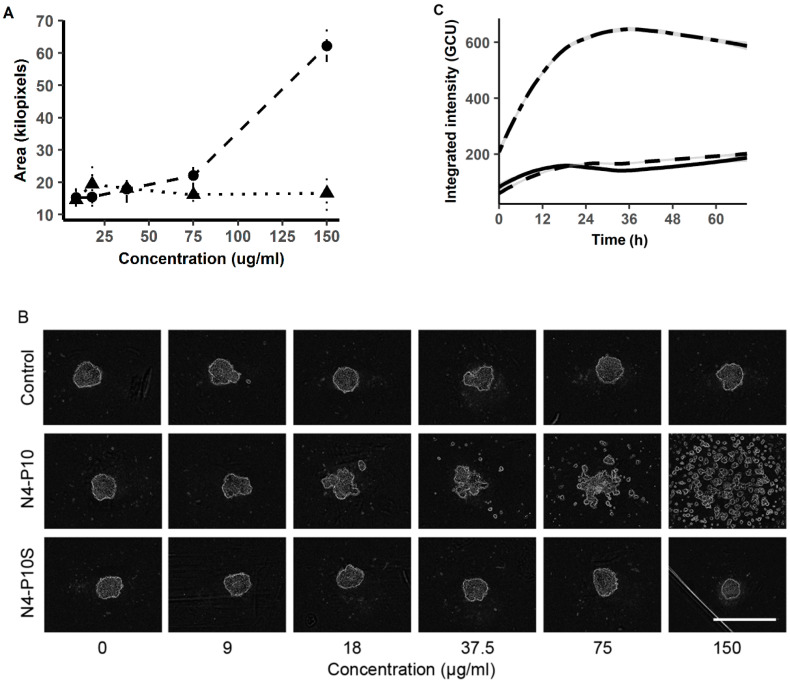
Nectin-4 peptide N4-P10 inhibits spheroid formation in a dose-dependent manner, and does not affect cell viability. (**A**) NIH:OVCAR5 cells were treated with the peptide N4-P10 (circle; dashed line) or a scrambled-sequence version of the peptide N4-P10 (N4-P10S; triangle, dotted line) at concentrations from 9 µg/mL to 150 µg/mL. Shown is the average spheroid area and SEM in kilopixels measured after 24 h of incubation. (**B**) Representative images of the cells after 24 h in the presence of the increasing concentrations of peptides N4-P10 and N4-P10S, or DMSO (control). Bar = 800 µm. (**C**) NIH:OVCAR5 cells treated concurrently with the IncuCyte^®^ Cytotox Green reagent and either 150 µg/mL peptide N4-P10 (dashed line), DMSO (solid line), or cytotoxic puromycin (two-dash line) were monitored on the IncuCyte^®^ S3 instrument for fluorescence intensity as a marker of cell death over the course of 72 h. Experiments were done in quadruplicate; shown is the average spheroid area and SEM (shaded line) at each 15 min time point over 24 h.

We also tested whether the ability of peptide N4-P10 to inhibit spheroid formation was dependent on the peptide concentration. NIH:OVCAR5 cells were incubated with peptide N4-P10 at 9–150 µg/mL for 24 h. At concentrations as low at 18 µg/mL, the peptide N4-P10 appeared to have a marginal effect of inhibiting spheroid formation, while the peptide N4-P10S had no effect compared to the DMSO control (Figure 5A,B). Increasing the concentration of peptide N4-P10 from 37.5 to 150 µg/mL caused the almost complete inhibition of spheroid formation, with the majority of the cells remaining separate from the others or in very loose aggregates (Figure 5A,B). In contrast, the peptide N4-P10S produced no quantifiable change in the formation of spheroids compared with the control (Figure 5A,B) at any concentration.

In order to ensure that the inhibitory effect of peptide N4-P10 was not the result of cytotoxicity, NIH:OVCAR5 cell death was measured as a function of integrated fluorescence intensity over time using IncuCyte^®^ Cytotox Green reagent. Compared to the negative control (DMSO) and positive control (cytotoxic puromycin), the cytotoxic effect of the peptide N4-P10 treatment was negligible; the cells treated with peptide N4-P10 were quantitatively indistinguishable from the untreated controls (Figure 5C), even after 72 h in the presence of peptide N4-P10. In contrast, the cells treated with the cytotoxic agent puromycin had essentially all died within 24 h (Figure 5C) as shown by the high fluorescence intensity.

### 2.5. Peptide N4-P10 Inhibits a Second Ovarian Cancer Cell Line from Forming Spheroids

**Figure 6:**
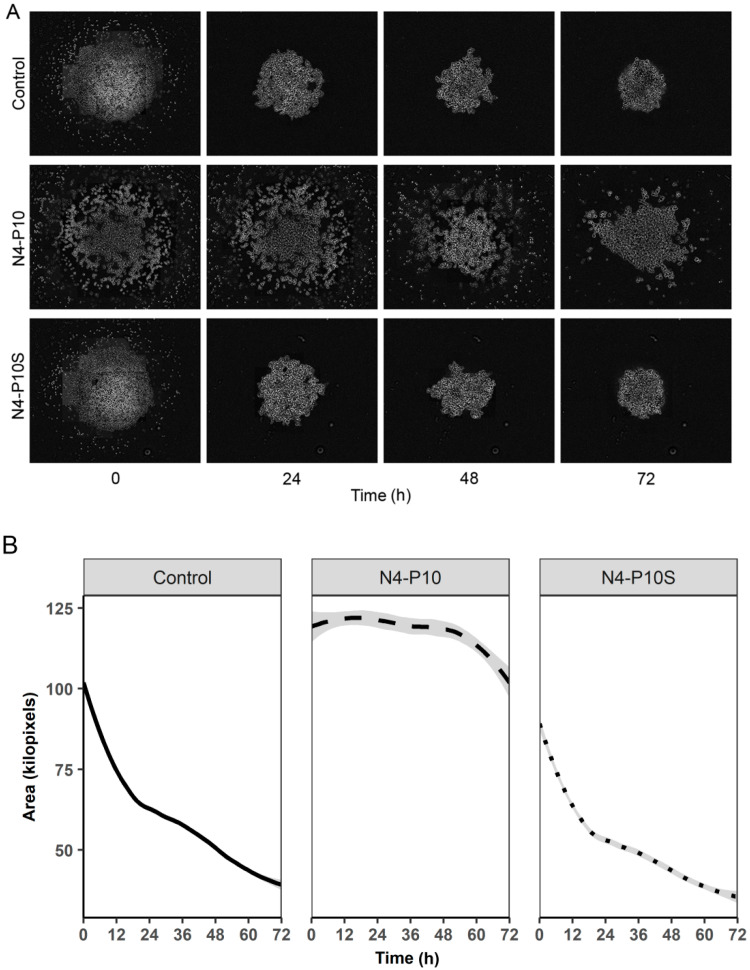
Peptide N4-P10 inhibits spheroid formation in CAOV3 ovarian cancer cells. DMSO (control) or 150 µg/mL of peptide N4-P10 or N4-P10S were added to 96-well round-bottom ultra-low attachment plates followed by the addition of CAOV3 cells. Spheroid formation was complete after 72 h. (**A**) Representative images of cells. The initial photographs (t = 0) were taken 10–25 min after the cells were plated, at which time the cells had gravitated to the bottom of the well and were initiating cell–cell attachments. Bar = 800 µm. (**B**) Spheroid size over time was measured using a CellProfiler^TM^ workflow. Experiments were done in quadruplicate; shown is the average spheroid area and SEM (shaded line) at each 15 min time point over 72 h. (Ave SEM +/− 3.78 kilopixels for Control; +/− 7.13 kilopixels for N4-P10 and +/− 3.50 kilopixels for N4-P10S). Time-lapse videos corresponding to these figures are included as Supplemental videos S8–S10.

In previous studies, we examined the role of Nectin-4 in the biological functions of ovarian cancer cells [14,15]. We showed that NIH:OVCAR5 cells form tight spheroid aggregates on flat-bottom low-attachment plates, while a second human ovarian cancer cell line, CAOV3, formed more loosely aggregated spheroids [15]. In this study, we sought to more comprehensively analyze the role of Nectin-4 in spheroid formation. Therefore, we also tested whether the peptide N4-P10 would have an inhibitory effect on the aggregation of CAOV3 cells. As was the case when we used the NIH:OVCAR5 cells, peptide N4-P10 inhibited the spheroid formation of CAOV3 cells (Figure 6; Supplemental video S8). CAOV3 cells treated with DMSO (control) or peptide N4-P10S formed aggregates over 24 h that contracted into more compact spheroids after 72 h (Figure 6A; Supplemental videos S9 and S10). When the size of the CAOV3 multicellular aggregates were measured (Figure 6B), the aggregates formed in the presence of peptide N4-P10 were not as tightly packed as the spheroids that were formed in the presence of peptide N4-P10S or the DMSO control, even after 72 h of incubation. These data demonstrate that the inhibitory effect of the peptide N4-P10 on spheroid formation can be recapitulated in multiple ovarian cancer cell lines with different capacities for spheroid formation.

## 3. Discussion

The formation of multicellular spheroids is a key component of disease progression in ovarian cancer, and may play a role in metastatic spread and chemoresistance. In this study, we screened twenty-one Nectin-4 peptides for their ability to inhibit spheroid formation in a real-time in vitro assay system that allowed us to accurately and reproducibly visualize and quantify early time points in the spheroid formation process. Notably, several of the Nectin-4 peptides inhibited spheroid formation to some extent, yet one peptide, N4-P10, caused the substantial inhibition of spheroid formation and was the focus of the majority of the experiments in this study. Peptide N4-P10 inhibited the formation of spheroids in a time and concentration-dependent manner for two human ovarian cancer cell lines, NIH:OVCAR5 and CAOV3. A scrambled version of the peptide N4-P10 did not have the inhibitory effect and peptide N4-P10 was not found to be cytotoxic. Taken together, these data suggest that the peptide N4-P10′s ability to prevent ovarian cancer cell aggregation could increase the efficacy of chemotherapy by maintaining cells as single cells or small aggregates.

In our previous analyses of ovarian cancer spheroids [15,16,17], several different methods were used to promote spheroid formation by making the tissue culture plastic of flat-bottom plates a “low-attachment” surface, e.g., by coating the wells with agarose, or the use of commercially prepared flat-bottom plates. When the NIH:OVCAR5 cells were seeded into these low-attachment flat-bottom environments, nearly all of the cells formed large, tightly compacted spheroids, while genetically modified cell lines that expressed reduced levels of Nectin-4 formed smaller aggregates amidst a large number of single cells [15]. Similarly, in our initial peptide screening we used flat-bottom ultra-low attachment plates and saw a pattern of spheroid formation in the cells treated with the Nectin-4 peptides that inhibited spheroid formation and resembled the Nectin-4 knockdown cells, i.e., many small spheroids in a background of single cells.

For the time-lapse experiments reported herein, we used round-bottom ultra-low attachment plates to monitor the spheroid formation at early time points and over an extended period of time, using time-lapse photography in IncuCyte^®^ instruments. In the round-bottom plates, NIH:OVCAR5 cells gravitated to the center of the wells and then formed a single, tightly compacted spheroid within 8 h. In contrast, the spheroids formed from N4-KD-VB9 cells in the round-bottom plates were slower to form the initial cell–cell attachments and the spheroids formed by 24 h were not as tightly compacted as the parental NIH:OVCAR5 cells. In the case of the cells treated with peptide N4-P10, multiple small aggregates formed in the round-bottom wells, which, after 24 h, encompassed an area that remained larger than the control spheroids.

The peptides that demonstrated the strongest inhibition of spheroid formation in our live cell imaging assay were among the same peptides with the ability to block ovarian cancer cell adhesion to Nectin-1 in a short-term binding assay [15]. These results suggest that the peptides were binding to proteins on the cell surface. In our previous study, peptides N4-P10 and N4-P22 both inhibited cell adhesion by approximately 50%; however, the inhibition of spheroid formation by these two peptides was more disparate, with peptide N4-P10 almost completely inhibiting spheroid formation. In contrast, peptide N4-P27 did not inhibit ovarian cancer cell adhesion to recombinant Nectin-1, but was found to inhibit spheroid formation in this study.

**Figure 7:**
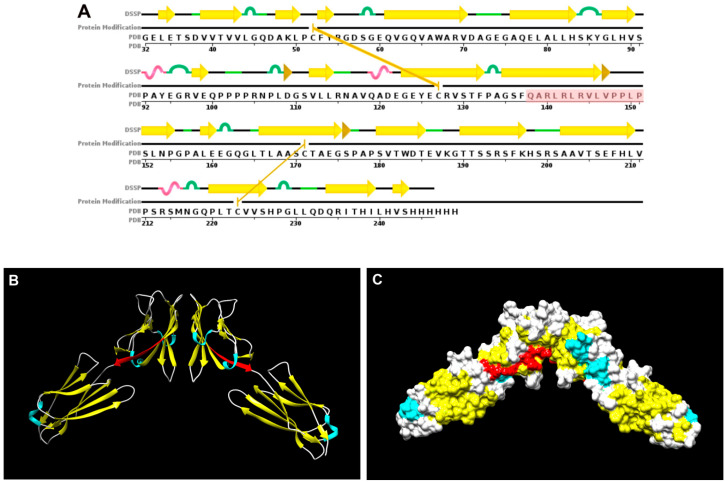
Molecular models showing the location of peptide N4-P10 in Nectin-4. The crystal structure of the IgV and IgC1 extracellular domains of human Nectin-4 were present in the Research Collaboratory for Structural Bioinformatics (RCSB) Protein Data Bank (PDB) (www.rcsb.org) as PDB ID: 4FRW [18] (**A**) The sequence chain view of the IgV and IgC1 domains modified from an image of Nectin-4 in the RCSB PDB, in which the amino acid sequence for the peptide N4-P10 is highlighted in red. Yellow arrows: beta strand; brown arrowheads: beta bridge; green loops: bend; pink loops: 3/10-helix; gold lines: cysteine bridges. (**B**) A 3-dimensional ribbon model of the dimerized IgV and IgC1 domains of Nectin-4. (**C**) A 3-dimensional surface model of the dimerized IgV and IgC1 domains of Nectin-4. In (**B**) and (**C**), the peptide N4-P10 is colored red, while the regions with structures are colored yellow (strands), blue (alpha-helix), and white (coil). The resolution of the original crystallographic study was between 20 and 3.5 angstroms, and the dimer interface shown in (**B**) and (**C**) is ~ 680 square angstroms [18].

Remarkably, none of the Nectin-4 peptide sequences that we found to interfere with spheroid formation in this study were shown to be important for Nectin-1:Nectin-4 interactions based on the protein crystal structure [18]. However, the sequence of peptide N4-P10, which we found to have the strongest inhibitory effect on spheroid formation, is immediately adjacent to the sequence of the Nectin-4 “F-strand” of the IgV domain that was shown to bind to a similar region of the Nectin-1 protein [18]. The amino acid sequence of the peptide N4-P10 resides in the area connecting the IgV domain with the IgC1 domain (Figure 7A). Three-dimensional modeling of the crystal structure of these two domains of Nectin-4 [18] indicates that the peptide N4-P10 is comprised of a beta strand (Figure 7A,B), and is exposed on the surface of Nectin-4 (Figure 7C). Perhaps rather than directly blocking the association between Nectin-1 and Nectin-4, the binding of peptide N4-P10 may act by decreasing the affinity of the interaction by changing the protein conformation. In this study, we observed a phenotypic resemblance between NIH:OVCAR5 cells that do not express Nectin-4 (N4-KD-VB9) and NIH:OVCAR5 cells that were treated with peptide N4-P10. In each case, the cell lines exhibited a delay in the formation of spheroids, resulting in spheroids that appeared to be less tightly aggregated; this delay may be attributed to Nectin-4 functioning as an initiator of cell adhesion [10].

Similarly, the reduction in spheroid formation by the peptide N4-P22, which comprises part of the second IgC domain of Nectin-4, could be due to a conformational change that affects the interaction between the IgV domains of Nectin-1 and Nectin-4. Alternatively, the peptide N4-P22 could be blocking the interactions between other proteins that are important for nectin function in spheroid formation. A region of Nectin-1 that is homologous to peptide N4-P22 was identified as binding to the FGF-receptor in the brain [19] and Nectin-4 binds to the prolactin receptor in mammary gland development [20] interacting through the cis-binding of the IgC2 domain [21].

In this study, peptide N4-P27 inhibited spheroid formation by almost forty percent compared to the control, although it had no effect on ovarian cancer cell adhesion to recombinant Nectin-1 in our previous study [15]. The amino acid sequence of peptide N4-P27 resides adjacent to the Nectin-4 transmembrane domain and contains the putative cleavage site for the proteases ADAM10 and ADAM17 [14,22]. While it is possible that the peptide N4-P27 was simply unable to recognize and bind to the recombinant Nectin-1 protein coating the microtiter plate [15], another possibility is that blocking the cleavage of the extracellular domain has novel effects in the longer time course of spheroid formation. The cleavage of the extracellular domain of nectins has the potential to negatively regulate cell adhesion, and in this manner, could initiate the dispersion or detachment of cancer cells from the primary tumor. Cleavage could also impart signaling functions by allowing the extracellular domain to bind in trans to growth factor receptors, as has been demonstrated for Nectin-1 and the FGF-receptor [19].

Previous work on spheroid formation in ovarian cancer cell lines described two general steps in spheroid formation, namely adhesion and contraction [23]. Our time-lapse imaging of spheroid formation suggests that Nectin-4 may function in both parts of this process. At the early time points (before ~6 h), peptides N4-P10 and N4-P22 appeared to inhibit cell–cell adhesion. Nectins have been shown to initiate intercellular adhesion during the assembly of adherens junctions [24]. After the initiation of cell adhesion by nectin family members, E-cadherin is recruited to the cell adhesion sites through association with proteins bound to the intracellular domain of the nectin molecules [24]. At later time points in spheroid formation (between 8–10 h for the NIH:OVCAR5 cells reported herein) the aggregates contracted into a tightly compacted spheroid. Our results with the NIH:OVCAR5 cell line show that this contraction phase was inhibited in cells that had Nectin-4 expression knocked down (N4-KD-VB9). The formation of compact spheroids is thought to depend on actinomyosin contractility [23], which could be mediated by the cytoplasmic region of Nectin-4 that is linked to actin filaments via afadin [10]. NIH:OVCAR5 cells treated with peptide the N4-P10 were primarily blocked at the adhesion stage. However, the peptide N4-P22 was not as effective at blocking cell adhesion, and at later time points the spheroids did contract, although they were not as compact as the controls. A similar lack of spheroid adhesion and contraction was observed for the CAOV3 cells treated with peptide N4-P10. However, the process of spheroid formation for the CAOV3 cells occurred more gradually over the course of 72 h, and even without peptide treatment, CAOV3 spheroids were not as tightly compacted as those formed by the NIH:OVCAR5 cells. Sodek et al. demonstrated that the ovarian cancer cell lines that form compact spheroids are more invasive than the loosely formed aggregates [23], suggesting that the inhibition of spheroid formation by Nectin-4 peptides could reduce ovarian cancer metastasis.

In addition to Nectin-4, other cell adhesion molecules, such as cadherins and claudin-4 have been implicated in spheroid formation and metastasis in ovarian cancer [16,25,26]. Targeting the cell adhesion molecules involved in spheroid formation could improve the sensitivity to chemotherapeutic agents. In colon, lung and breast cancer cells, antibodies against E-cadherin disrupt spheroids, sensitizing cells to chemotherapy by increasing the intracellular drug accumulation [27]. Similarly, using peptides to block the formation of laminin-induced spheroids resulted in the improved efficacy of cisplatin in ovarian cancer cells [28]. In breast cancer cells, where Nectin-4 promotes anchorage-independent growth, antibodies against Nectin-4 have been shown to block the growth of tumor cell implants in mice [29]. These data, together with our current study showing that Nectin-4 peptides can starkly inhibit spheroid formation, suggest that using peptides to inhibit cell adhesion and spheroid formation may be a novel approach to increase response to chemotherapy in ovarian cancer.

## 4. Materials and Methods

### 4.1. Cell Lines

The human ovarian cancer cell lines NIH:OVCAR5 (Judah Folkman, Harvard University, Boston, MA, USA) [30] and CAOV3 (Robert C. Bast Jr., MD Anderson Cancer Center, Houston, TX, USA) were received in 1995 and viably stored in liquid nitrogen. After thawing, the cells were grown in a complete medium (RPMI 1640 media containing 10% fetal bovine serum (FBS) for NIH:OVCAR5 cells or DMEM containing 10% FBS for CAOV3) at 37 °C in a humidified incubator with 5% CO_2_. Cell line identity was verified by single nucleotide polymorphism (SNP) analysis (MD Anderson Cell Authentication Core Facility, Houston, TX, USA) and the cells were tested for mycoplasma contamination. NIH:OVCAR5 Nectin-4 shRNA knockdown cells, N4-KD-VB9, which express no detectable Nectin-4 protein, have been previously described [15].

### 4.2. Peptide Synthesis and Purification

Peptides of 14 amino acids in length from the extracellular domain of Nectin-4 and scrambled-sequence control peptides were synthesized by Aapptec (Louisville, KY, USA) as previously described [15]. A scrambled version of peptide N4-P10 was used as a control peptide and is called N4-P10S. The amino acid sequences of the peptides are listed in Table 1. All the peptides were synthesized with a free amino terminus and an amide at the carboxy terminus, purified by high-pressure chromatography, and the sequences were verified by mass spectrometry to be >95% pure. The peptides were dissolved in dimethyl sulfoxide (DMSO; Sigma-Aldrich, St. Louis, MO, USA) at a concentration of 50 mg/mL, aliquoted, and stored at −80 °C.

### 4.3. Real-Time Spheroid Formation Monitored by Time-Lapse Videos in IncuCyte^®^ Zoom Instrument

Confluent monolayers of ovarian cancer cell lines (NIH:OVCAR5, N4-KD-VB9, or CAOV3) were detached from the tissue culture flasks using 0.05% Trypsin (ThermoFisher Scientific, Waltham, MA, USA) and resuspended at a concentration of 1.0 × 10^5^ cells/mL in complete media. Each well of a 96-well round-bottom ultra-low attachment plate (Corning, Corning NY, USA) was seeded with 50 ul of the resulting suspension. In most cases, the experiments were conducted with eight technical replicates at each condition. The plates were then placed in the gantry of the IncuCyte^®^ Zoom (Sartorius, Ann Arbor, MI, USA) live cell analysis system and incubated at 37 °C in 5% CO_2_ for 24–72 h, as indicated. Photographs were taken at ~15 min increments for a total of no less than 48 h for the NIH:OVCAR5 cell lines or up to 72 h for the CAOV3 cell line. The initial photographs (*t* = 0) were taken 10–25 min after the cells were plated, at which time the cells had gravitated to the bottom of the well and were initiating the cell–cell attachments. The resulting footage was analyzed to determine the change in the area of the spheroid aggregates over time. The images were binarized to black and white, and the sum of the area of the small aggregates (black objects) in pixels was determined via the CellProfiler^TM^ workflow described below. Each image was normalized to the same dimensions, so that the areas could be compared.

### 4.4. Screening Nectin-4 Peptides for Effect on NIH:OVCAR5 Spheroid Formation

For the initial screening of the twenty-one Nectin-4 peptides, 50 ul of the peptides at 300 µg/mL (for a final working concentration of 150 µg/mL peptide per well) in complete media were added to 96-well flat-bottom ultra-low attachment plates (Corning, Corning, NY, USA). DMSO was used as a negative control. Fifty microliters of NIH:OVCAR5 cells at a concentration of 1.0 × 10^5^ cells/mL in complete media were added to each well. The plates were then incubated at 37 °C in 5% CO_2_ for 48 h, with photographs taken manually at 24 and 48 h on a Nikon^®^ TE200 microscope. A single screening experiment was conducted with 8 technical replicates at each condition to determine which peptides had the most significant effect on spheroid formation.

Experiments were then repeated using round-bottom ultra-low attachment plates in which the cells were added to the wells containing the peptides at a final concentration of 150 µg/mL. The plates were placed in the IncuCyte^®^ Zoom instrument for 24-48 h of live cell imaging, as described above. Experiments with the peptides selected for further study were performed in triplicate, with 8–16 technical replicates at each treatment condition.

### 4.5. Dose Dependence of Peptide N4-P10 on Spheroid Formation

NIH:OVCAR5 cells were added to wells containing from 9 to 150 µg/mL of peptide N4-P10 or peptide N4-P10S, or DMSO control, and then the formation of spheroids was monitored via the IncuCyte^®^ Zoom instrument. The resulting footage was analyzed with the CellProfiler^TM^ pipeline described below.

### 4.6. Cytotoxicity Testing of Peptide N4-P10

The cytotoxicity of the peptides N4-P10 and N4-P10S on the NIH:OVCAR5 cells was monitored for 72 h as a function of fluorescence intensity over time after treatment with 250 nM IncuCyte^®^ Cytotox Green in the IncuCyte^®^ S3 instrument (Sartorius, Ann Arbor, MI, USA). As a positive control for cytotoxicity, 2 µg/mL puromycin was used.

### 4.7. CellProfiler^TM^ Analysis

A simple four-step CellProfiler™ (open source, Broad Institute, www.cellprofiler.org) pipeline was used to delineate the biologically relevant image contents and to quantify the area of spheroids over time. As part of the CellProfiler™ workflow, cellular edges were enhanced using the Sobel method, the images were converted to binary via Otsu method thresholding, and the area of occupied pixels as a function of overall image size was recorded [31]. The resulting values were exported for analysis and visualization in the R statistical programming language. The values were averaged and the mean with the standard error of the mean (SEM) was determined and graphed. Experiments were routinely performed with eight replicates for each condition, and repeated a minimum of two times on separate days. Since many photographs were taken during the course of each experiment (approximately every 15 min) and the SEM was very small, it is difficult to see the small error bars in the graphs. For this reason, the values for each experimental SEM were included in the figure legends.

### 4.8. Three-Dimensional Modeling of Nectin-4

The crystal structure of the IgV and IgC1 extracellular domains of human Nectin-4 were present in the Research Collaboratory for Structural Bioinformatics (RCSB) Protein Data Bank (PDB) (www.rcsb.org) as PDB ID: 4FRW [18]. The location of the peptide N4-P10 in the Nectin-4 molecule was visualized using the crystal structure [18] and UCSF Chimera (http://www.cgl.ucsf.edu/chimera/).

## 5. Patents

Patent 15/595,604 is pending in the United States Patent and Trademark Office entitled, “Inhibitors of cell adhesion” which documents the Nectin-4 peptides that were used in this study.

## Figures and Tables

**Table 1:** Amino acid (AA) sequences and the domains of Nectin-4 peptides.

Name	Peptide AA Sequence	AA Number	Domain
N4-P3	YRGDSGEQVGQVAW	54-67	IgV
N4-P4	AWARVDAGEGAQEL	66-79	IgV
N4-P5	ELALLHSKYGLHVS	78-91	IgV
N4-P6	VSPAYEGRVEQPPP	90-103	IgV
N4-P7	PPPRNPLDGSVLLR	102-115	IgV
N4-P8	LRNAVQADEGEYEC	114-127	IgV
N4-P10	QARLRLRVLVPPLP	138-151	IgV/IgC1
N4-P10S	RPVQLALPRLVRPL	NA	
N4-P11	LPSLNPGPALEEGQ	150-163	IgC1
N4-P13	EGSPAPSVTWDTEV	174-187	IgC1
N4-P14	EVKGTTSSRSFKHS	186-199	IgC1
N4-P15	HSRSAAVTSEFHLV	198-211	IgC1
N4-P16	LVPSRSMNGQPLTC	210-223	IgC1
N4-P17	TCVVSHPGLLQDQR	222-235	IgC1
N4-P19	AEASVRGLEDQNLW	246-259	
N4-P20	LWHIGREGAMLKCL	258-271	IgC2
N4-P21	CLSEGQPPPSYNWT	270-283	IgC2
N4-P22	WTRLDGPLPSGVRV	282-295	IgC2
N4-P23	RVDGDTLGFPPLTT	294-307	IgC2
N4-P25	SNEFSSRDSQVTVD	318-331	IgC2
N4-P26	VDVLDPQEDSGKQV	330-343	
N4-P27	SGKQVDLVSASV	339-350	Adjacent to membrane

## Supplementary Materials

Supplementary Materials can be found at https://www.mdpi.com/1422-0067/21/13/4637/s1.

## Abbreviations

3D	3 Dimension
N4-P10	Nectin 4-Peptide 10
N4-P10S	Nectin 4-Peptide 10 Scrambled Sequence
RT-PCR	Reverse Transcriptase-Polymerase Chain Reaction
NIH:OVCAR5	National Institutes of Health: Ovarian Carcinoma 5 Cell Line
CAOV3	Carcinoma Ovarian 3 Cell Line
shRNA	Short Hairpin RiboNucleic Acid
N4-KD-VB9	Nectin 4-Knockdown-Version B9
SEM	Standard Error of the Mean
Ig	Immunoglobulin-Like Domain
TM	Transmembrane Domain
AA	Amino Acid
DMSO	Dimethyl Sulfoxide
ADAM10	Disintegrin and Metalloproteinase Domain-Containing Protein 10
ADAM17	Disintegrin and Metalloproteinase Domain-Containing Protein 17
FGF	Fibroblast Growth Factor
FBS	Fetal Bovine Serum
SNP	Single Nucleotide Polymorphism
